# “Internal carotid artery-type cervical spondylosis” is not supported as an independent subtype: a perspective on diagnostic classification and clinical differentiation

**DOI:** 10.3389/fneur.2026.1842860

**Published:** 2026-08-27

**Authors:** Tianjun Zhai, Yeping Chen, Wei Feng, Hui Zhang

**Affiliations:** 1 School of Acupuncture, Moxibustion and Tuina (School of Rehabilitation Medicine), Anhui University of Chinese Medicine, Hefei, China; 2 The Second Rehabilitation Hospital of Shanghai, Shanghai, China

**Keywords:** carotid disease, cerebrovascular disease, cervical spondylosis, cervicogenic dizziness, diagnostic classification, differential diagnosis, internal carotid artery

## Abstract

Cervical spondylosis is a common degenerative disorder, yet its diagnostic boundaries may be inappropriately expanded when nonspecific symptoms such as dizziness coexist with age-related cervical imaging changes. This Perspective evaluates the hypothetical concept of “internal carotid artery-type cervical spondylosis” and whether it qualifies as an independent clinical subtype. Using an adapted validity–utility framework, the proposed entity was assessed according to five criteria: a specific anatomical relationship, a reproducible pathophysiological mechanism, a recognizable clinical phenotype, supportive imaging or ancillary evidence, and independent diagnostic utility. Unlike the vertebral artery, the cervical internal carotid artery has no consistent anatomical relationship with routine degenerative cervical structures. Rare bony–carotid conflicts may occur, but these are anomaly-driven vascular lesions and do not establish a general degenerative subtype. Nonspecific dizziness does not localize pathology to the internal carotid artery, whereas focal anterior-circulation symptoms are better explained by established carotid or cerebrovascular disorders. Clinically, transient monocular visual loss, aphasia, hemiparesis, focal cortical deficits, or new unilateral head or neck pain with partial Horner syndrome or cranial nerve findings should prompt urgent vascular or stroke-oriented evaluation. Current evidence therefore does not support “internal carotid artery-type cervical spondylosis” as an independent subtype. Maintaining clear diagnostic boundaries may reduce mislabeling and improve patient safety.

## 1 Introduction

Cervical spondylosis is a common degenerative disorder encountered in orthopedics, rehabilitation medicine, neurosurgery, and neurology. Its best-established clinical syndromes include axial neck pain, cervical radiculopathy, and degenerative cervical myelopathy (1). Diagnostic difficulty arises when common age-related cervical imaging changes coexist with nonspecific complaints such as dizziness, tinnitus, head discomfort, or a vague sense of “insufficient cerebral blood supply.” Because structural degeneration is prevalent and these symptoms have heterogeneous causes, coexistence alone does not establish causation (2).

The issue is particularly relevant in the Chinese clinical context. Traditional Chinese classifications provided a broad clinical vocabulary for cervical presentations and were incorporated into historical Chinese diagnostic frameworks. Vertebral-artery and sympathetic types were sometimes used when patients presented with dizziness, tinnitus, nausea, palpitations, or other symptoms without clear nerve-root or spinal-cord involvement (3, 4). The 2018 Chinese expert consensus subsequently adopted a more cautious structure, retaining cervical, nerve-root, and spinal-cord types while grouping vertebral-artery and sympathetic presentations within “other types” (3).

International literature likewise emphasizes better-defined entities such as axial neck pain, cervical radiculopathy, and degenerative cervical myelopathy (5, 6). Dizziness associated with neck symptoms is generally discussed as “cervicogenic dizziness” or “cervical dizziness,” concepts that remain controversial because no single mechanism or confirmatory test has been established (7, 8). Contemporary interpretations increasingly emphasize altered cervical proprioception and disturbed sensorimotor integration, while reserving hemodynamic explanations for selected, demonstrable vascular circumstances (2, 8).

The phrase “internal carotid artery-type cervical spondylosis” is not recognized in current Chinese consensus or mainstream international classification. This article therefore does not present it as a documented, widespread terminological error. Instead, the phrase is used as a deliberately hypothetical and pedagogical boundary case: if artery-related naming were extended from the historically discussed vertebral-artery category to the internal carotid artery, would the proposed entity satisfy the requirements of a clinically valid and useful subtype?

The article first reviews current classification trends, then applies an explicit validity-and-utility framework, addresses both ordinary anatomy and rare osteovascular exceptions, and finally presents a practical red-flag pathway for distinguishing degenerative cervical presentations from carotid and cerebrovascular disease. The purpose is not to deny that rare cervical bony structures can interact with carotid vessels, but to determine whether such exceptions justify a general degenerative subtype and to reduce the risk that vascular disease is mislabeled as cervical spondylosis.

## 2 Current perspectives in China and internationally

### 2.1 Perspective from China

Traditional Chinese classifications provided a broad clinical vocabulary for cervical presentations and were incorporated into historical Chinese diagnostic frameworks. Vertebral-artery and sympathetic types were especially attractive when patients presented with dizziness, tinnitus, nausea, palpitations, or other symptoms without clear nerve-root or spinal-cord involvement (3, 4). This framework had practical historical value, but it also permitted heterogeneous symptom clusters to be linked to cervical degeneration without a sufficiently specific mechanism or diagnostic boundary.

The 2018 expert consensus reflects a shift toward more conservative subtype recognition (3). By placing vertebral-artery and sympathetic presentations within “other types,” the consensus did not necessarily eliminate every possible cervical contribution to dizziness; rather, it reduced the taxonomic status of categories whose mechanisms, phenotype, and confirmatory criteria were less secure. This change is consistent with a broader evidence-based preference for diagnoses that guide reproducible evaluation and management.

### 2.2 International perspective

Internationally, cervical spondylosis is usually organized around clinical syndromes with demonstrable neural or mechanical correlates: axial neck pain, cervical radiculopathy, and degenerative cervical myelopathy (5, 6, 9). These entities are not defined by imaging alone. Their diagnostic strength derives from concordance among symptoms, examination findings, anatomical level, and the exclusion of competing disorders.

Cervicogenic dizziness remains a diagnosis of exclusion and is often discussed in relation to cervical proprioceptive dysfunction rather than a presumed global reduction in cerebral blood flow (2, 7, 8). When the internal carotid artery is suspected, the appropriate framework is carotid disease, cervical artery dissection, transient ischemic attack, or stroke, with evaluation directed toward vascular morphology and cerebral ischemia (10).

### 2.3 Implication for the present analysis

The absence of “internal carotid artery-type cervical spondylosis” from recognized classifications is not, by itself, proof that the concept is invalid. Conversely, it should not be portrayed as an omitted term that clinicians commonly use unless such circulation is documented. The present analysis therefore adopts a narrower claim: the phrase is a hypothetical extension that can be tested against explicit criteria for subtype validity and clinical utility.

This approach also clarifies the paper’s clinical purpose. The relevant question is not merely whether the internal carotid artery lies in the neck, but whether labeling carotid-related symptoms as a cervical spondylosis subtype improves diagnostic accuracy, investigation, or treatment. If the label does not add value beyond established vascular categories—and may delay their recognition—it should not be adopted even if occasional anatomical contact can be demonstrated.

## 3 An adapted framework for evaluating a proposed clinical subtype

Kendell and Jablensky (11) distinguished the validity of a diagnosis from its clinical utility. Although their discussion arose from psychiatric nosology, the underlying distinction is cross-disciplinary: a category should have evidentiary coherence, and it should improve communication, prediction, or management. The present article does not import psychiatric criteria wholesale. It adapts the general validity–utility distinction to a structural/vascular problem and operationalizes it using principles also applied in neurological subtype appraisal (12).

Five requirements are used. First, the proposed target structure should have a sufficiently specific and characteristic anatomical relationship to degenerative cervical changes. Second, a coherent and reproducible pathophysiological mechanism should link those changes to the proposed manifestations. Third, the subtype should show a recognizable symptom-and-sign profile rather than a loose collection of nonspecific complaints. Fourth, imaging or other ancillary tests should support the proposed causal relationship, not merely demonstrate coincidental abnormalities. Fifth, the category should possess independent diagnostic utility by changing differential diagnosis, testing, prognosis, or treatment.

Established cervical syndromes illustrate these requirements. In cervical radiculopathy, foraminal compromise or local irritation affects an identifiable nerve root and produces a broadly concordant pattern of radiating arm pain, sensory change, weakness, and reflex abnormality (9). Degenerative cervical myelopathy similarly combines a relevant structural lesion with long-tract and hand-function findings that carry clear management implications (6). Neither diagnosis is established by degenerative imaging alone; clinical–radiological concordance remains essential.

**Figure 1:**
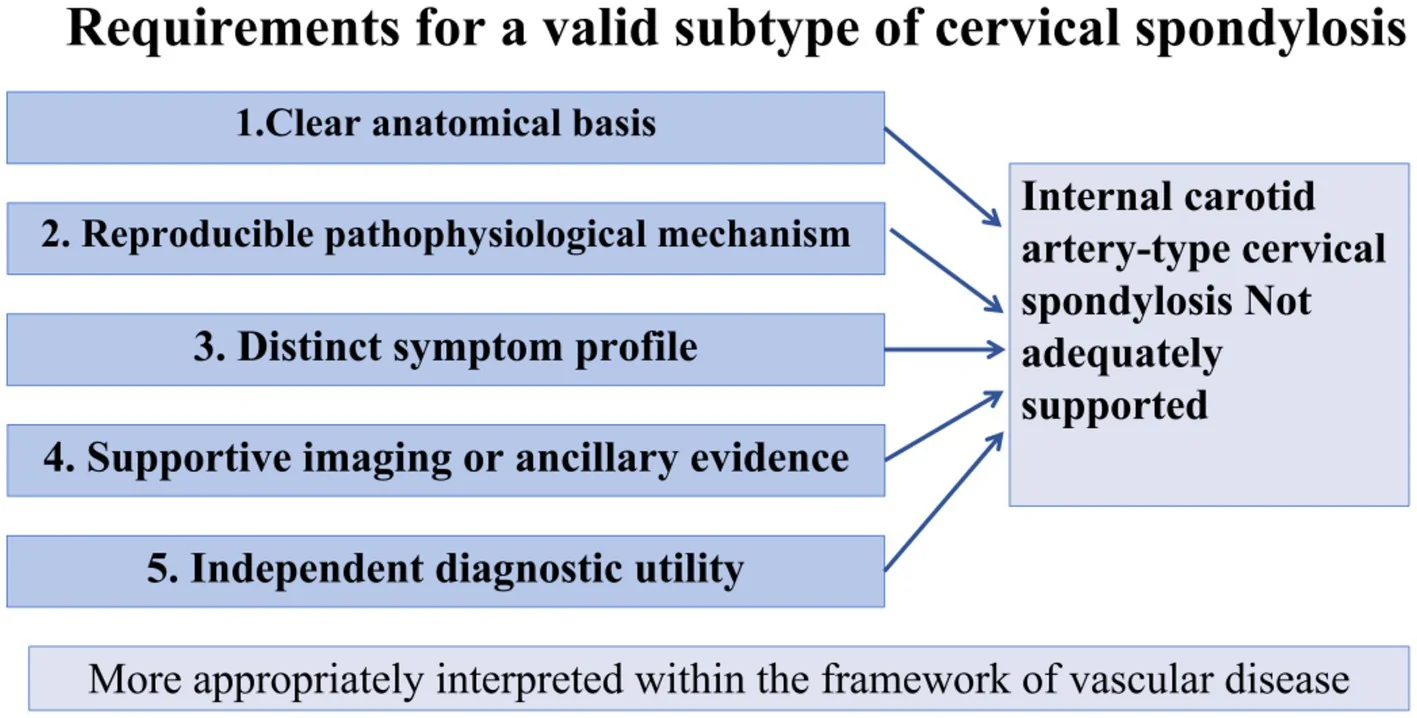
Analytical framework for evaluating whether a proposed subtype of cervical spondylosis qualifies as an independent clinical entity.

A hypothetical internal carotid subtype would therefore require more than the simultaneous presence of cervical degeneration and carotid disease. It would need a characteristic degenerative lesion–artery relationship, a reproducible causal pathway, a distinct phenotype, confirmatory vascular evidence attributable to that lesion, and a clinical advantage over existing carotid and cerebrovascular diagnoses. Figure 1 summarizes this analytical framework.

## 4 Anatomical and pathophysiological appraisal

### 4.1 The vertebral artery: a direct but clinically contested relationship

After arising from the subclavian artery, the vertebral artery typically enters the transverse foramen at C6, ascends through the cervical transverse foramina, courses around the atlas, and then enters the cranial cavity (13). This route creates a direct and reproducible spatial relationship with the cervical osseous and ligamentous structures. Osteophytes, congenital variants, instability, or extreme rotation can therefore plausibly influence the vessel in selected patients.

That anatomical plausibility does not automatically validate a broad “vertebral artery type” of cervical spondylosis. Rotational vertebral-artery syndrome and osteophyte-related vertebral stenosis are uncommon, require dynamic or vascular confirmation, and are better understood as specific vascular compression syndromes than as explanations for all dizziness associated with cervical degeneration (14, 15). The vertebral artery thus provides an instructive distinction between anatomical possibility and diagnostic generalizability.

### 4.2 The internal carotid artery: the ordinary cervical course

The internal carotid artery arises at the carotid bifurcation and ascends toward the skull base within the carotid sheath, accompanied by the internal jugular vein, vagus nerve, and connective tissue (16). Unlike the vertebral artery, it does not normally traverse the cervical transverse foramina or another repetitive osseous canal. Its cervical segment usually occupies a soft-tissue neurovascular corridor rather than a space in which routine uncovertebral osteophytes, foraminal narrowing, or disc-height loss predictably contact the artery.

Accordingly, the internal carotid artery does not ordinarily share a comparably direct structural relationship with common degenerative cervical changes. This wording is intentionally not absolute: the neck contains variable bones, ligaments, and vessels, and rare contact phenomena can occur. The classification question is whether a stable and characteristic relationship exists across patients with ordinary cervical spondylosis, not whether any cervical bony structure can ever contact a carotid vessel.

### 4.3 Rare osteovascular conflicts and the Naito Gomi case

Rare anterior-circulation osteovascular conflicts are well documented, including carotid injury associated with an elongated styloid process, hyoid-related compression, and other abnormal bony relationships (17). Naito Gomi and colleagues reported an 87-year-old woman with a carotid web in the common carotid artery adjacent to an elongated C6 transverse process; three-dimensional angiography suggested that repeated mechanical injury and dissection might have contributed to the lesion (18). This case is an important counterexample to any categorical statement that cervical bone and the carotid system can never interact.

The case does not, however, establish an “internal carotid artery-type cervical spondylosis.” First, the lesion was located in the common carotid artery rather than the cervical internal carotid artery. Second, the proposed mechanism involved an elongated transverse process and a focal, anomaly-driven contact, not the ordinary pattern of disc degeneration, uncovertebral arthrosis, or foraminal narrowing that defines cervical spondylosis. Third, the evidence derives from a single case with a proposed rather than experimentally reproduced causal pathway. Finally, the patient’s lesion was investigated through ultrasonography, angiography, computed tomography, and magnetic resonance angiography and remained conceptually within a vascular/stroke framework (18). The case therefore strengthens the need to recognize rare osteovascular conflicts while also illustrating why they should retain specific vascular names rather than be generalized into a degenerative cervical subtype.

### 4.4 Mechanistic implications

The dominant pathological processes of cervical spondylosis are disc degeneration and secondary osseous, ligamentous, and soft-tissue changes. Their established consequences are local mechanical pain, nerve-root irritation or compression, and spinal-cord compression (19, 20). These mechanisms are coherent because the affected tissues lie within or immediately adjacent to the degenerative motion segment.

For dizziness associated with neck symptoms, contemporary discussion increasingly emphasizes disturbed cervical afferent input and sensorimotor integration; vascular mechanisms are reserved for selected conditions with demonstrable arterial compromise (8, 21). This distinction prevents a plausible but unproven vascular narrative from being applied to nonspecific dizziness simply because cervical imaging shows degeneration.

Internal carotid pathology is usually driven by atherosclerosis, plaque instability, thrombosis, embolism, dissection, fibromuscular dysplasia, or other vessel-wall disorders (10, 22–24). Rare mechanical conflicts may contribute when an elongated or abnormally positioned bone repeatedly contacts the artery (17, 18, 24), but such mechanisms are lesion-specific and vascular. They do not demonstrate that routine cervical degeneration is a defining cause of internal carotid disease.

The mechanistic conclusion is therefore qualified rather than categorical: ordinary cervical spondylosis does not provide a characteristic or reproducible basis for an internal carotid subtype, whereas rare bony–arterial conflicts should be diagnosed according to the specific vascular lesion and anatomical anomaly that are actually demonstrated.

## 5 Clinical differential diagnosis and red-flag pathway

**Figure 2:**
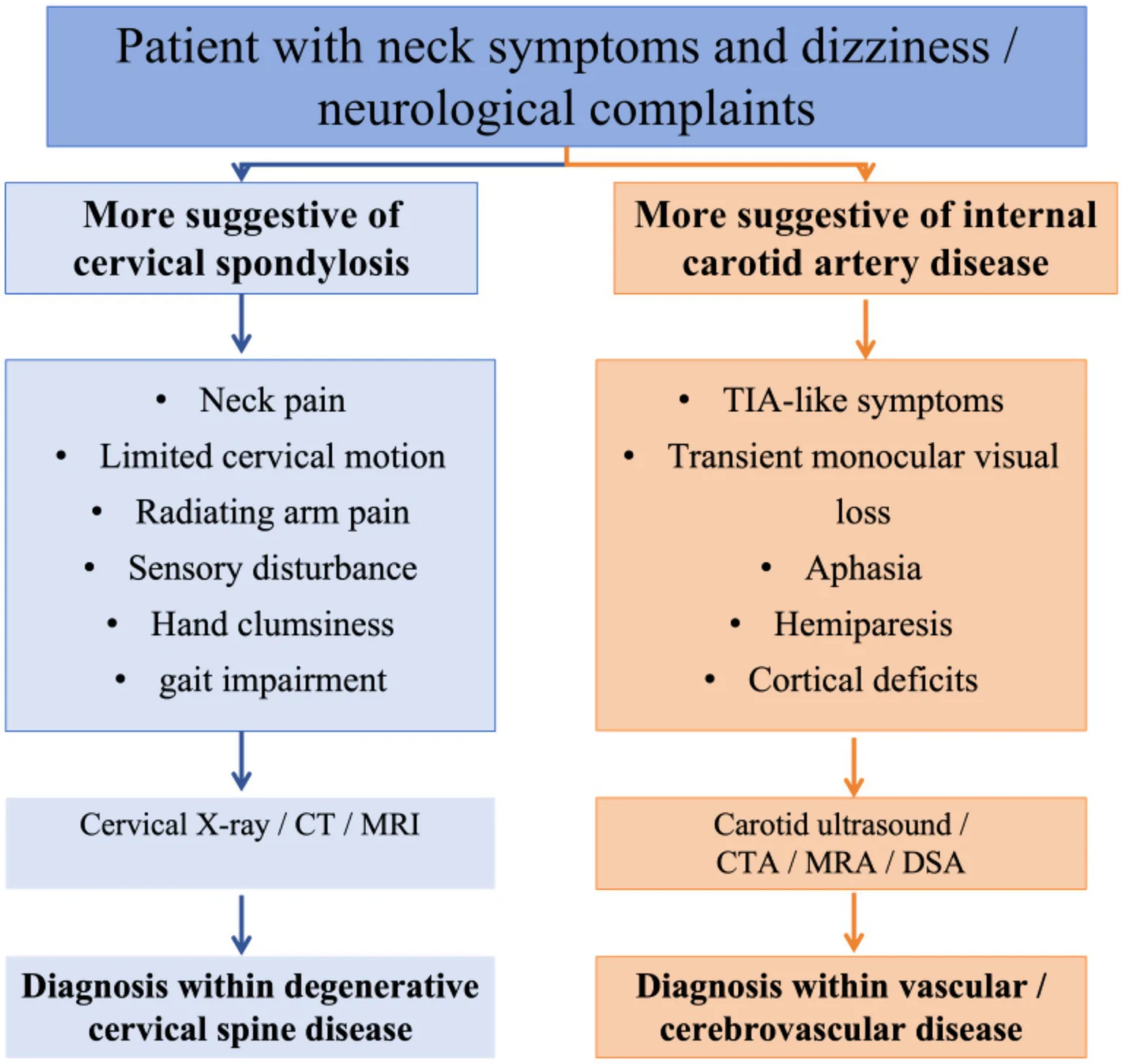
Diagnostic distinction between cervical spondylosis and internal carotid artery-related vascular disorders.

The principal clinical contribution of this analysis is to prevent diagnostic mislabeling. Neck pain and dizziness commonly coexist, and degenerative findings are frequent on cervical imaging. A cervical abnormality should not become the default explanation for dizziness or neurological symptoms unless the clinical pattern and examination support a cervical syndrome and relevant competing causes have been considered. Figure 2 provides a simplified diagnostic distinction; the subsections below expand the red flags and practical sequence.

### 5.1 Features favoring degenerative cervical spine disease

A cervical-spondylosis explanation is strengthened by a mechanically coherent pattern: neck pain or stiffness related to posture or movement; dermatomal arm pain, paresthesia, or sensory loss; myotomal weakness or reflex change; or signs of degenerative cervical myelopathy such as hand clumsiness, gait imbalance, hyperreflexia, and long-tract dysfunction (1, 6, 9, 19, 20). Cervical imaging may support the diagnosis when the level and side of neural compression correspond to the clinical findings (25).

Imaging alone is insufficient. Disc degeneration, osteophytes, and canal or foraminal narrowing may be incidental, particularly in older adults. Similarly, reproduction of nonspecific dizziness by neck movement does not by itself prove a vascular mechanism; head movement can provoke vestibular, proprioceptive, orthostatic, or anxiety-related symptoms. When dizziness is the dominant complaint without a concordant radicular, myelopathic, or clearly mechanical syndrome, the clinician should widen rather than narrow the differential diagnosis (2, 7, 8, 21).

### 5.2 Features favoring carotid or cerebrovascular disease

Symptoms that suggest anterior-circulation ischemia include transient monocular visual loss, aphasia or other language disturbance, unilateral face or limb weakness or numbness, neglect, and other focal cortical deficits (10, 23). Sudden onset, transient stereotyped episodes, or persistent focal deficits should trigger an emergency stroke or transient ischemic attack pathway rather than outpatient attribution to cervical spondylosis. Dizziness in isolation is nonspecific and is not a characteristic internal-carotid syndrome, but dizziness accompanied by focal neurological findings materially changes the level of concern.

Cervical internal-carotid dissection deserves particular attention when new or unusual unilateral head, facial, or neck pain is accompanied by partial Horner syndrome, cranial-nerve palsy, pulsatile tinnitus, or cerebral ischemic symptoms. A history of recent minor trauma or cervical manipulation may increase suspicion but is not required, and the absence of conventional atherosclerotic risk factors does not exclude dissection (24). A carotid bruit or substantial vascular risk burden may support investigation for atherosclerotic carotid disease, although neither is sufficiently sensitive to rule disease in or out by itself (10, 23).

### 5.3 A practical diagnostic sequence

The first step is triage. Sudden focal neurological deficit, transient monocular blindness, aphasia, hemiparesis, or a dissection-type pain syndrome with localizing signs requires urgent neurological and vascular assessment. The immediate priority is not to decide whether the patient also has cervical degeneration, but to avoid delaying time-sensitive stroke evaluation.

The second step is a focused history and examination. Clinicians should separate focal from nonfocal symptoms; characterize onset, duration, recurrence, and triggers; document vascular risk factors and recent trauma; and perform a neurological examination that includes cranial nerves, visual fields, language, limb power and sensation, coordination, gait, reflexes, and signs of Horner syndrome. A musculoskeletal examination should assess cervical range of motion, pain provocation, radicular pattern, and myelopathic signs without using symptom reproduction alone as proof of vascular compression.

The third step is mechanism-directed testing. The choice among cervical radiography, computed tomography, and magnetic resonance imaging should be guided by the clinical presentation, suspected neural or structural abnormality, and the presence or absence of red flags (25). Carotid ultrasonography, computed tomography angiography, or magnetic resonance angiography is appropriate when the history, examination, or risk profile raises concern for carotid stenosis, dissection, or another vascular lesion; digital-subtraction angiography is reserved for selected diagnostic or interventional circumstances (10, 23, 24). Dynamic vascular imaging may be required when symptoms are position-dependent and a specific osteovascular conflict is suspected (14, 15, 17).

The final step is diagnostic labeling. Coexisting cervical degeneration and carotid disease should usually be recorded as separate conditions unless a specific causal osteovascular relationship is demonstrated. Using established vascular terminology—such as carotid stenosis, cervical carotid dissection, carotid web, or an anatomically defined osteovascular conflict—communicates the relevant mechanism and directs appropriate treatment more effectively than an umbrella label of “internal carotid artery-type cervical spondylosis.”

## 6 Synthesis against the five criteria

When the hypothetical subtype is evaluated against the adapted framework, it does not satisfy the required threshold. Its ordinary anatomical basis is weak because the cervical internal carotid artery is not predictably coupled to routine degenerative motion-segment changes. Its mechanism is not reproducible because the dominant causes of internal carotid disease are vascular, while mechanical cases depend on uncommon and individually demonstrable anomalies. Its phenotype is not distinct because nonspecific dizziness does not localize the internal carotid artery, whereas focal anterior-circulation symptoms already map to established cerebrovascular diagnoses. Its ancillary testing is not subtype-specific because positive findings are interpreted as carotid stenosis, dissection, web, or another vascular lesion. Finally, it lacks independent utility because the proposed label does not improve management beyond existing vascular categories and may instead delay them.

This synthesis does not deny rare bony–carotid interactions. It draws a classification boundary between (1) a common degenerative disease with established neural syndromes, (2) controversial dizziness presentations requiring careful exclusion of other causes, and (3) rare, anatomically specific osteovascular conflicts that belong within vascular and cerebrovascular medicine. Maintaining this boundary is both more accurate and more clinically useful.

## 7 Limitations and future directions

This Perspective is a structured conceptual analysis rather than an empirical prevalence study, systematic review, or validation cohort. It does not establish how often clinicians or patients may spontaneously use the hypothetical phrase, and the present analysis makes no claim that the term is in routine circulation. The conclusions are based on recognized classifications, anatomy, pathophysiology, illustrative vascular literature, and diagnostic utility.

Research on cervicogenic dizziness continues to evolve. Future work should clarify which combinations of symptoms, examination findings, and sensorimotor tests identify a clinically meaningful cervical contribution; quantify how often cervical degeneration is incorrectly assigned as the cause of dizziness; and develop prospective pathways that reduce delayed recognition of vestibular, migraine, cerebrovascular, and systemic disorders. Rare osteovascular conflicts also warrant registries or multicenter case series that distinguish reproducible syndromes from isolated anatomical coincidences.

## 8 Conclusion

“Internal carotid artery-type cervical spondylosis” is best treated as a hypothetical classification exercise, not as an established diagnostic term. Current evidence does not support it as an independent subtype because ordinary cervical spondylosis lacks a characteristic relationship to the internal carotid artery, no reproducible degenerative mechanism defines internal carotid pathology, and the relevant symptom patterns and investigations are already better represented by vascular and cerebrovascular diagnoses.

Rare mechanical interactions between cervical bony structures and the carotid system are clinically important, but they are anomaly-driven osteovascular conflicts and should be named and managed according to the specific arterial lesion. The practical message is therefore to avoid attributing nonspecific dizziness or focal neurological symptoms to cervical degeneration by default, recognize vascular red flags promptly, and select imaging according to the suspected mechanism. This diagnostic discipline preserves the core boundaries of cervical spondylosis while improving patient safety.

## Data Availability

The original contributions presented in the study are included in the article/supplementary material, further inquiries can be directed to the corresponding authors.
